# 

**Published:** 1892-03

**Authors:** 


### Society Notes.

### ANNOUNCEMENT AND INVITATION.

*To the Members of the Texas State Medical Association, and the Regular Medical Profession of Texas:*

The Texas State Medical Association announces that its twenty-fourth annual meeting will be held at Tyler, commencing Tuesday, April 26, 1892, and requests the honor of your presence.

Tyler is accessible from all parts of the State, and is reached by the Tyler branch of the I. & G. N. R. R., so that the meeting is sure to be largely attended. Ample accommodations will be secured by the Committee of Arrangements, and no member should fail to be present.

The value of effective Professional organization is too apparent to need further comment. The Association now has a membership of nearly five hundred, and should have as many more. Its scientific work is divided into nine sections, in each of which papers of interest and value are apt to appear. At each meeting there should be nine annual reports. The President's Address, Dr. W. H. Wilkes, Waco; report of the Chairman of the Section on Practice of Medicine, Dr. T. J. Bell, Tyler; report of the Chairman of the Section on Obstetrics, etc., Dr. J. T. Musick, Pittsburg; report of the Chairman of the Section on Surgery, etc., Dr. C. H. Wilkinson, Galveston; report of the Chairman of the Section on Medical Jurisprudence, etc., ——; report of the Chairman of the Section on State Mecicine, etc., Dr. J. L. Cunningham, Fort Worth; report of the Chairman of the Section on Gynecology, Dr. T. H. Nott, Goliad; report of the Chairman of the Section on Ophthalmolgy, etc., Dr. R. C. Hodges, Galveston; report of the Chairman of the Section on Dermatology, Dr. R. W. Knox, Houston; report of the Chairman of the Section on Microscopy, etc, Dr. J. W. McLaughlin, Austin.

Are you a member of the Association? If so, will you not add to the interest of the meeting by reading a paper upon some subject in which you are specially interested? Kindly send your name at once, and the subject of the paper you will read, to the General Secretary, at Galveston, in order that its title may be published.

Are you a member of the regular profession, and not a member of the Association? Then a most cordial invitation is extended to you to be present at the coming meeting at Tyler, and there present your application for membership. You will find it to your personal and professional advantage to identify yourself with the work of the State Medical Association.

